# Supplementary material for: Are carbon emissions trading and green financial instruments synergistic? -Comprehensive quantitative research based on content analysis
Source: PLoS One. 2024 Mar 7;19(3):e0298601. doi: 10.1371/journal.pone.0298601 (PMC10919601; doi:10.1371/journal.pone.0298601)
Supplement: S1 Data — (ZIP) [file pone.0298601.s001.zip › Data/地级市Z_F工作报告.zip!/╔╜╬≈/╠1⁄2╘¡╩╨/└┤╫╘╣1⁄2╓┌║┼í╛┬φ┐╦╩2╛▌═°í┐_2014.docx]

各位代表：

现在，我代表市人民政府向大会报告工作，请予审议，并请政协委员和其他列席人员提出意见。

一、2013年工作回顾

2013年，在省委、省政府和市委的正确领导下，全市认真贯彻落实党的十八大精神，积极应对挑战，奋力克难攻坚，转型综改全面推进，圆满实现全年经济社会发展主要预期目标，在全省年度目标责任考核中名列第一。

——经济运行稳中向好。完成地区生产总值2412.87亿元，增长8.1%；规模以上工业增加值770.94亿元，增长10.1%；固定资产投资1670.74亿元，增长26.5%；社会消费品零售总额1281.46亿元，增长13.5%；公共财政预算收入247.33亿元，增长14.7%；城镇居民人均可支配收入24000元，增长11%；农民人均纯收入11288元，增长12%；CPI平均上涨3.1%。

——调结构转方式成效初显。一、二、三产投资分别增长44.5%、21.9%和28.4%。省、市两级重点工程完成投资1765.43亿元。新兴接替产业投资和增加值双超传统产业，分别占到全市规模以上工业的53.2%和54.3%，其中亿元以上新兴接替产业项目105个、完成投资227.58亿元。服务业投资占全市投资的比重提高0.9个百分点，增加值占地区生产总值的54.8%。江铃重汽发动机、中天信安防科技、T800级高端碳纤维等重点项目进展顺利。都市现代农业取得新进展。国内首个反映产地煤炭市场价格的“太原指数”正式上线。武宿综合保税区封关运行，对外开放的格局进一步扩大。

——城市基础设施建设扎实推进。新建改造并州路、府东府西街等城市主次干道105条，轨道交通2号线试验段开工。中环快速交通主线全长48.46公里，全程架设高架桥20.29公里，下穿通道16座，上跨道路、河道桥梁17座，大型互通立交8座，跨汾河特大异型桥1座，总计建设里程104.61公里，标志着太原步入立体交通时代。智慧城市建设迈出新步伐。生态太原建设力度加大，全年造林35.94万亩，城市绿化253万平方米，建成区绿化覆盖率、绿地率分别提高0.81和0.79个百分点。全省造林绿化现场会在我市召开，实施“生态新政”经验得到充分肯定。

——省城环境质量明显提升。拆除分散燃煤锅炉543台，集中供热扩网2148万平方米。228台常年运行燃煤锅炉实施“煤改气”。完成11个城中村整村拆除，拔掉黑烟囱11663根。太化氯碱、晋阳选煤厂等232家污染企业实施关停、淘汰和搬迁。城南污水处理厂建成投运。汾河水库纳入国家重点支持江河湖泊动态名录，全市地下水位止降回升1.08米，集中式饮用水源地水质达标率保持100%。加大对汽车尾气排放的治理，淘汰3.6万辆老旧车辆。全年PM2.5达标率54.5%，优良天数162天，实现省城环境质量改善明显见效的目标。

——民生社会事业进一步改善。全年完成保障性住房投资89亿元，基本建成2.96万套，分别增长49.43%和214%。5000户农村危房改造任务全面完成，2.3万贫困人口脱贫。城镇登记失业率控制在3.35%，城镇新增就业10.7万人。出台破产改制国有企业职工安置政策，积极稳妥解决职工养老、医疗保险问题。教育、卫生保障水平不断提高。公共自行车服务点覆盖四分之三以上建成区。“一元菜”惠民活动深受好评。成功举办2013太原国际马拉松赛。扎实推进平安省城建设，依法稳妥处置突发事件，严厉打击各类犯罪，群众安全感进一步提升。加强食品药品市场整治。狠抓安全生产，严格落实“两个主体”责任，全年生产经营性事故起数下降3.81%，死亡人数下降6.61%。

——政府自身建设不断加强。自觉接受人大及其常委会的监督，提请市人大常委会制定修订《太原市客运出租汽车服务管理条例》等7件地方性法规。积极支持人民政协履行职能，坚持重大事项向民主党派通报和协商制度。194件人大代表建议和603件政协提案全部办复。认真贯彻落实中央八项规定，坚决反对“四风”，“三公”经费明显下降。“向人民汇报、请人民评议”等活动更加深入，有效推动政府作风转变。 “两集中、两到位”行政审批制度改革取得明显成效，全省深化行政审批制度改革现场会在我市召开。

这些成绩的取得，是省委、省政府和市委正确领导，市人大、市政协监督支持，全市人民共同努力的结果。在此，我代表市人民政府向全市人民，向驻并部队、武警官兵，向中央、省属单位，向各民主党派、工商联、无党派人士、人民团体，向各位人大代表、政协委员，向所有关心支持太原改革发展的海内外朋友们，表示崇高的敬意和衷心的感谢！

在肯定成绩的同时，也要清醒地看到，我们发展道路上还有不少困难和问题。主要是：经济稳中向好基础还不牢固，支撑转型跨越发展的好项目、大项目不多；改革开放力度不大，经济增长内生动力不足，下行压力增大；大气和水污染问题仍然突出，省城环境质量好转任务艰巨；城市基础设施建设滞后，民生改善的压力加大；社会信用体系不健全，影响稳定的潜在矛盾依然存在；公务员队伍的执行力和专业化水平不足，群众观念不强，廉政建设需进一步加强。民之所望，施政所向；病之所在，治之所要。我们将牢记责任使命，凝心聚力，扎实有效解决问题，决不辜负人民的厚望。

二、2014年工作安排

2014年，我们面临的形势依然错综复杂，有利条件和不利因素并存。世界经济复苏的可能性增大，世界银行预测全球经济增长速度有望达到3.2%左右。我国全面深化改革开放的号角已经吹响，将进入历史性的新阶段，必将为经济社会发展注入强大动力。发展是解决所有问题的关键，牢牢抓住经济建设这个中心，保持合理的经济增长速度，是中央的大政方针。我们要紧紧把握可以大有作为的重要战略机遇期，加快调结构、转方式，充分发挥转型综改试验先导区的作用，实现转型跨越发展的新突破。

今年政府工作的总体要求是：高举中国特色社会主义伟大旗帜，以邓小平理论、“三个代表”重要思想、科学发展观为指导，全面贯彻落实党的十八届三中全会精神，学习贯彻习近平总书记系列重要讲话精神，坚持稳中求进、改革创新，加快一流的新兴产业基地、一流的自主创新基地和一流的现代宜居城市建设，着力保障改善民生，有效增强社会活力，切实维护和谐稳定，深入开展党的群众路线教育实践活动，为建设一流省会城市，为全省办好“两件大事”，为实现“中国梦”而努力奋斗。

经济社会发展的主要预期目标是：地区生产总值增长9%左右，规模以上工业增加值增长11%左右，服务业增加值增长9%，固定资产投资增长21%，社会消费品零售总额增长14%左右，公共财政预算收入增长9%左右，城镇居民人均可支配收入增长10%左右，农民人均纯收入增长12%以上，居民消费价格涨幅控制在3.5%左右，城镇登记失业率控制在4%以内，城镇新增就业10.5万人。

约束性指标是：万元地区生产总值综合能耗下降3.5%，单位工业增加值用水量和二氧化硫、化学需氧量、氮氧化物、氨氮、烟尘和工业粉尘排放量完成省下达任务，PM2.5年均浓度值下降5%。

实现上述目标任务，必须把握发展大势，因势而谋，应势而动，顺势而为，把发展的强大动力和内需的巨大潜力释放出来，着力推进转型综改试验先导区建设。重点抓好以下六个方面的工作：

（一）以改革开放为强大动力，释放更多改革红利

改革是今年政府工作的首要任务。要按照中央和省统一部署，以经济体制改革为重点，全面深化各领域改革。要从群众最期盼的领域改起，从制约经济社会发展最突出的问题改起，从社会各界能够达成共识的环节改起，力求取得实质性进展和突破。

深化行政体制改革，进一步简政放权。推进政府职能整合，最大限度地解决职能交叉和分散问题，用政府权力的“减法”换取市场和企业活力的“加法”。凡是公民、法人或其他组织能够自主决定，市场竞争机制能够有效调节，行业组织或中介机构能够自律管理的事项，政府都要退出；凡是可以采用事后监管和间接管理的事项，政府一律不设置审批；凡是中央、省下放给地方的行政审批事项，一律接转下放、一放到位；凡是中央和省取消的行政审批事项，一律放给市场、放给社会。深化投资审批制度改革，充分落实企业投资自主权，推进投资创业便利化、高效化。推进国有企业改革，发展混合所有制经济。实施工商登记制度改革，落实认缴登记制，由先证后照改为先照后证，由企业年检制度改为年报公示制度。创新改革思路，利用大数据支撑，建成区范围内建设项目取消覆压矿产、地质灾害评估、详规评审等前置性条件，减少重复浪费和时间损失。

加强事中事后监管，坚持放管并重。实现责任和权力同步下放，放活和监管同步到位，做到放而不乱、疏而不漏，放开市场这只“看不见的手”，用好政府这只“看得见的手”。对市场主体，“法无禁止即可为”；对政府，“法无授权不可为”。进一步深化“两集中、两到位”行政审批制度改革，建立权力清单制度，推进“负面清单”管理模式，明确立即办、承诺办、限期办事项，优化流程，缩短时间，阳光政务，从严监督。加快社会信用体系建设，推进政府信息共享，对违背市场竞争原则和侵害消费者权益的企业建立黑名单制度，让失信者寸步难行，让守信者一路畅通。大力推进电子政务建设，实现信息化支撑下的精细化管理。

完善政府治理能力的基础制度建设，用制度管权、管钱。要把工程建设项目招投标、政府采购、国有土地使用权和矿业权出让等公共资源交易，纳入规范化、法制化轨道，让市场发挥决定性作用。推进建设用地批后监管，建立土地集约节约利用评价考核体系。严格公共资金管理和监督，把政府所有收支纳入预算管理，做到收入一个“笼子”，预算一个“盘子”，支出一个“口子”，防止公共资金碎片化，体现集中财力办大事的原则。政府预算和决算要向社会公开，部门预算要逐步公开到基本支出和项目支出，所有财政拨款的“三公”经费都要公开，所有公共资金、公共资产、公共资源都要接受审计和社会监督，打造“阳光财政”。进一步加强国库资金管理，建立财政结转、结余资金定期清理制度，提高资金使用绩效。公款姓公，一分一厘都不能乱花；公权为民，一丝一毫都不能私用。

（二）以调结构转方式为主攻方向，推动产业提质增效升级

科技创新是产业升级的关键。要抓住新一轮科技革命的历史机遇，推动科技与经济紧密结合、信息化和工业化深度融合。认真评估大数据对经济社会运行所带来的革命性影响，科学制定未来发展规划。强化与北大、清华、中科院等院校、科研单位产学研用深度合作，大力引进“千人计划”等高端创新人才，巩固和发展院士工作站。支持各类创新要素向企业集聚，促进科技成果转化，推动创新引领发展。大力发展战略性新兴产业，加速催化中天信安防科技、罗克佳华物联网、众人科技等高新技术产业成长壮大。发挥好清华科技园、大数据产业园等新兴产业创业创新平台作用，实现创新主体聚集、创新产业聚变。强化项目牵动引领，发挥投资关键作用，加快江铃整车、发动机研发制造项目建设，推动太原轨道交通铁路装备园等20个重大项目达产达效。加大传统产业技术改造和整合重组力度，提升产业素质和竞争优势。

现代服务业是未来经济持续健康发展的潜力所在。推进服务业发展提速、比重提高、水平提升，是太原转型跨越战略的重要支撑。要发挥省会城市品牌优势，大力发展总部经济和楼宇经济，推进汾酒集团总部、山西国际金融中心建设，推进华润万象城、万达综合体、中海、绿地、万科CBD中心、润恒冷链农产品物流中心等服务业项目建设。互联网电子商务是现代服务业划时代的变革，要鼓励创新主体积极运用互联网思维，带动传统产业转型，实现商业模式创新，加快电子商务示范城市建设。加大科技创新风险投资支持力度，加快组建太原农商行，完善政银企“助保贷”合作机制，发挥政府资金“四两拨千斤”之力，为企业创新和中小企业发展“施肥增养”。

把培育新的区域经济带作为推动发展的战略支撑。融入环渤海经济圈，加大招商引资力度，加强与京津地区区域性合作。发挥好高新区、经济区、民营区和不锈钢园区招商引资、产业发展主体功能区作用，加快阳曲、清徐产业新城规划布局和开发建设，推进古交煤电、煤化一体化发展，着力打造娄烦省城饮用水源地良好生态环境的品牌优势。

（三）以一流省会城市为目标，强化城市基本建设

城市是人类文明的结晶，城镇化是现代化的必由之路。建设生态宜居的美丽太原，是全市人民的共同心愿。要按照中央城镇化工作会议的精神，“生产空间集约高效，生活空间宜居适度，生态空间山清水秀”，“让居民望得见山，看得见水，记得住乡愁”，凸显太原“三面环山、一水中分”的大自然格局，做好山水特色城市的谋篇布局。

加强城市道路改造和建设。新建滨河东路南延、阳兴大道、迎宾桥，续建太行路南延、南中环东延、双塔路南延，改造建设路、解放南路、长治路、南沙河路等23项道桥工程，总计建设里程110公里，总投资170亿元。继续优化提升中环快速道路交通体系，配套完善周边路网，放大环路快速效应。积极推进轨道交通2号线建设，启动滨河西路南延前期准备，加大小街小巷整治力度。城市道路建设集管线入地、立面改造、违建拆除、棚户区改造、拆迁安置、社会动员于一体，是一场深刻的社会变革，要秉承多留遗产、少留遗憾的理念，坚持规划、设计、建设、管理的高标准。

加快新区发展。推进产城一体为特色的汾东新区建设，借山西科技创新城发展之契机，顺势而为，协同发展。推进以长风商务区和晋阳湖为中心的晋阳新区建设，现代与传统融合，生态与文化比翼。加强以太原南站为中心的新区建设，高点起步，连片改造，形成集高铁、公交、长途客运、地铁于一体的交通大枢纽，打造代表太原现代气派的靓丽窗口。继续创建国家“公交都市”，更新公交车1200台，新增公交专用道20公里，启动新能源汽车试运行工作。继续增加公共自行车投放。推进健康城市建设，完善以汾河公园为载体的健康休闲体系。加强智慧城市建设，实施便民惠民“一卡通”，强化以信息化为支撑的城市管理，创新方法，加大力度，建管并重，提升省会城市的管理水平。

打好省城环境质量改善攻坚战。落实“三年大见成效”目标措施。关闭东山电厂、美锦矸石电厂等污染企业，加大对二电厂等重污染企业周边环境的治理力度。以电力、冶金、焦化、水泥等重污染行业为重点，实施百家企业对标一流整治工程。推进集中供热全覆盖，新增嘉节燃气电厂、二电厂、太钢余热利用集中供热3000万平方米，开工建设太古远距离、大温差供热工程。淘汰分散燃煤锅炉600台左右，改造既有供热面积2000万平方米以上，减少燃煤90万吨。加快能源清洁化步伐，常年运行燃煤锅炉必须在九月底全部置换为燃气锅炉，拒不落实的要承担关门停业的代价。大力淘汰老旧车辆和黄标车，严控机动车尾气面源污染。开工建设32万吨晋阳污水处理厂，清徐两个1500吨垃圾焚烧电厂、餐厨垃圾处理厂，清徐、阳曲两个秸秆电厂，源头根治环境污染。实施南沙河、虎峪河、九院沙河雨污分流和源头截污治污工程。严格执行施工现场绿色标准，坚决控制扬尘污染。加快城中村和棚户区改造，从根本上消除居民煤灶污染，全面推进城乡清洁工程。完成24个城市周边村燃气清洁替代，把农民从污染能源的重负中解放出来。铁腕治污，铁规治污，严肃查处各类环境污染案件。三县一市要同步落实责任，为全市环境质量好转做出贡献。

生态环境是最大的公共产品，事关人民群众的幸福感。加强精细化管理，建立生态环境的红线和生态补偿制度。继续实施“生态新政”，加大东西两山生态绿化投入力度，建立大规模、高质量、美景观的生态绿色屏障。启动晋祠泉域复流工程，开工建设8平方公里的晋阳湖、2平方公里的植物园，完善提升中环50公里的绿色长廊，实施建成区绿色覆盖系统工程，见空插绿，拆墙透绿，建园植绿，力争建成区绿化覆盖率、绿地率均提高0.6个百分点，人均公园绿地提高0.3个百分点。

（四）以都市现代农业为抓手，推动城乡一体化发展

坚持城乡统筹发展。以工业化理念谋划发展，强化龙头带动、基地联动、市场驱动。大力推进都市现代农业园区建设，加大马铃薯青薯9号等新品种、新技术示范推广。加快宝迪肉制品加工、蒙牛奶业、九牛牧业等农产品龙头企业建设和规模化扩张，带动养殖业基地持续良性发展，形成市场牵龙头、龙头带基地、基地联农户协同互哺发展的共赢格局。鼓励企业家领办创办农业企业，为农业发展注入新的生产力要素，增强农业产业发展后劲。强化润恒、美特好等农产品物流中心的市场驱动力，引领设施农业规模化发展，支持三县一市新增设施蔬菜基地7000亩，推进阳曲百万只养羊工程，带动全市“一县一业”纵深发展。

大力改善农村人居环境。坚持因地制宜，尊重农民意愿，突出农村特色，建设美丽乡村。继续推进农村危房改造，提高农村饮水安全标准。依托农村田园风光，发展农家乐等乡村旅游，增加农民收入。创新扶贫开发方式，探索金融支持“三农”的措施和办法，推进百企千村产业扶贫开发工程和移民并村致富工程，建立农民增收的长效机制，实现2.3万贫困人口脱贫。

积极推进农村改革。坚持和完善农村基本经营制度，培育专业大户、家庭农场、农民合作社、农业企业等新型农业经营主体，积极开展土地确权登记试点工作，引导承包土地经营权有序流转，发展多种形式适度规模经营，赋予农民更多财产权利。认真组织第十届村民委员会换届工作。

（五）以唐风晋韵为文脉特色，彰显省城文化影响力

文化是城市的灵魂。一流省会城市，需要有一流文化的支撑。

加强社会主义核心价值体系建设。习近平总书记指出：“一个国家的文化软实力，从根本上说，取决于其核心价值观的生命力、凝聚力、感召力”。要继续培育和弘扬太原“包容、尚德、崇法、诚信、卓越”的城市核心价值观，凝魂聚气，强基固本，提升文化软实力。文化繁荣，内容为王，创意至上，精品取胜。大力推进文化精品创作，生动展示现代、文明、发展的太原形象。加强博物馆、美术馆、图书馆等公共文化场馆建设。精心组织好第十二届全国美术作品雕塑展，提升和扩大太原文化的影响力。

加强历史文化名城保护。城市形成是历史的积累和沉淀，城市需要传承记忆。有2500年建城史的古老太原，要有代表历史资格的老城、老街、老屋。9平方公里的明清府城，要重点保护文庙、崇善寺片区，天主教堂、拱极门片区，文瀛湖、五一路片区，钟楼街、靴巷片区等四大块文物遗存相对集中的历史街区。坚持以点扩面、以面连片，积少成多、渐成气候。四大片区要落实最严格的保护措施，坚决禁止任何破坏性的建设行为。“往者不可谏，来者犹可追”。我们必须承担起保护和传承历史文化的重任，不能愧对历史、愧对先人。今年重点实施钟楼街历史街区保护工程，让掩藏在混乱建筑中的历史文化遗存重现珍贵的文化价值和商业价值。

加快发展文化旅游产业。突出板块表现、气候吸引、服务引领。要把晋祠和天龙山，太山龙泉寺、店头村，蒙山和连理塔寺庙群，崛山围山和窦大夫祠、多福寺、净因寺等四大板块的山水寺庙文化，进一步优化提升和保护发展，形成大山水、大文化、大景区、大旅游的产业格局。精心保护双塔寺大景区，完善景区功能，放大双塔效应。加快太化工业遗址创意产业发展，组织好国际大学生“废旧金属材料雕塑创作月”活动，打造具有国际影响力的城市新名片。

乡村文明是中华文明史的主体，耕读文明是我们的软实力。明太原县城作为农耕文明载体，要创新保护理念，在晋阳古城考古发掘的基础上，将田园风光与文化遗存相结合，创造农耕文明保护复兴的新范例。加大对青龙镇、土堂村等古村落保护的力度，让农耕文明的乡愁代代相传。

（六）以“五有”保障为基本要求，着力做好民生改善工作

民惟邦本，本固邦宁。政府工作的根本目的，是让人民群众过上好日子。要下大力气切实解决好“五有”等基本民生保障问题，不断提高人民生活水平和质量。

就业是民生之本。把稳定就业作为经济发展的优先目标，优化创业就业环境，以创新引领创业，以创业促进就业，开发更多就业岗位，解决高校毕业生就业问题，引导和鼓励大学生创业就业。落实城镇就业困难人员帮扶措施，确保零就业家庭至少有一人就业。发挥劳动力市场中介组织作用，实现需求和就业的有效对接。

努力解决好住房问题。居者有其屋，人们才有资格获得尊严。构建以政府为主提供基本保障、以市场为主满足多层次需求的城镇住房供应体系。今年再开工建设5万套安置保障住房，在两年内使拆迁户全部安置，把拆迁改造作为改变人生的新机遇，实现“征收谁、改变谁、造福谁”的惠民目标。支持企业和社会建设人才公寓，为引进和留住人才创造良好环境。严格执行统一政策和标准，加快棚户区和城中村改造步伐，对龙堡、东社、西寨、木厂头等13个城中村进行整村拆除，实现城市升级和人民宜居互利共享。推广邻里中心，集购物场所、老年活动、文化娱乐、社区服务、幼儿教育于一体，满足宜居、乐居的功能要求，创造幸福家园。

推进教育卫生资源均衡化、标准化、优质化，满足学有所教、病有所医的基本需求。加快五中、一外、二外、十二中、成成中学等10所名校双十二轨新校建设，完成二十七、五十三、六十中等10所学校改扩建工程，强制性规范配建幼儿园、小学，满足宜居城市基本服务半径。加快中心医院、人民医院、妇幼保健院等10所新院建设，加大公立医院改革力度，规范配建社区医院，建立便民利民的健康服务体系。加强与名校、名院合作，引进人才和管理；推进教育和医疗信息化，充分利用全国优质教育和医疗资源，大幅提高教育和医疗服务水平。大力发展职业教育，整合资源，提升质量。继续做好人口和计划生育工作。

健全完善社会保障体系。加大“五险”统一征缴力度，稳步提高统筹层次和保障水平。积极稳妥解决好破产企业、劳服企业、集体企业职工的养老和医疗保险问题，运用好低保等社会救助政策。开工建设政府主导的养老中心和儿童福利院，扩大城市社区居家养老试点，鼓励社会力量参与养老事业和残疾人救助等社会福利事业，支持慈善事业发展，让老有所养、幼有所爱的阳光普照大地。深化收入分配制度改革，强化企业职工工资调控，促进农民增收，进一步提高城乡居民收入。做好民族宗教工作。加强国防后备力量建设，推进双拥工作。

安全重于泰山。全面落实企业安全生产主体责任和政府安全生产监管责任，完善党政同责、一岗双责、齐抓共管的责任体系，加大安全生产指标考核权重，实行安全生产和重大安全生产事故风险“一票否决”；深入开展安全隐患有奖举报活动，完善安全隐患发现受理、处置问责制度；开展煤矿、非煤矿山、燃气、危险化学品、道路交通等专项治理行动，坚决杜绝重特大安全事故发生。健全和完善市、县、乡食品药品监管体系，加强信息化建设，用最严格的监管、最严厉的处罚、最严肃的问责，确保全市人民“舌尖上的安全”。积极创新社会治理体制，继续推进天网治安工程。扎实做好信访工作，加强法律援助，建立畅通有序的民意诉求表达渠道和矛盾纠纷排查调处机制，及时化解、妥善处置社会矛盾，最大限度地预防和减少各类不稳定因素。

各位代表，做好今年的政府工作，一分部署，九分落实。要结合党的群众路线教育实践活动，加强政府自身改革建设。按照国家治理体系和治理能力现代化的要求，推进目标管理，对标一流，严格考评，建设法治政府、创新政府、廉洁政府、务实政府，增强政府的公信力和执行力。要践行“三严三实”，严以修身、严以用权、严以律己，谋事要实、创业要实、做人要实。作风建设上行下效，行胜于言。“打铁还需自身硬”，“风行于上，俗成于下”。各级领导干部都要以己正立信、以廉政树威、以务实兴业，努力为人民提供优质高效服务。

要深入贯彻依法治国基本方略，自觉接受人大及其常委会的监督，接受人民政协的民主监督，认真听取人大代表、政协委员、民主党派、工商联、无党派人士和各人民团体的意见，提高依法治市、依法行政的自觉性。深入开展“向人民汇报、请人民评议”、“为人民群众办实事、解难事”和窗口单位行业“贴近群众、服务群众”等活动，自觉接受人民群众和社会舆论监督。加大政务公开力度，加强行政监察，纠正部门和行业不正之风。深入推进反腐倡廉制度建设，从严查处腐败案件。

各位代表，行进在改革开放新的伟大征程中，光荣与梦想召唤着我们，责任和使命激励着我们。让我们紧密团结在以习近平同志为总书记的党中央周围，在省委、省政府和市委的坚强领导下，以踏石留印、抓铁有痕的作风，敢于担当、勇往直前的精神，立党为公、公权为民的情怀，为实现率先转型跨越发展、建设一流省会城市而努力奋斗！
